# Supplementary material for: High resolution data modifies intensive care unit dialysis outcome predictions as compared with low resolution administrative data set
Source: PLOS Digit Health. 2022 Oct 11;1(10):e0000124. doi: 10.1371/journal.pdig.0000124 (PMC9931257; doi:10.1371/journal.pdig.0000124)
Supplement: S1 Table — (PDF) [file pdig.0000124.s001.pdf]

S1 Table. The eICU High Resolution Variables. The baseline characteristics of the high resolution variables for the patients in the eICU database.

Variable	Total cohort (n=8822)	No dialysis (n=8,095)	Dialysis (n=727)	p-value
**Laboratory tests, mean (SD)**				
Sodium (mEq/L)	138.70 (6.07)	138.81 (6.06)	137.48 (6.16)	<0.01
Potassium (mEq/L)	4.18 (0.74)	4.15 (0.72)	4.48 (0.89)	<0.01
Bicarbonate (mEq/L)	22.68 (6.09)	22.99 (6.03)	19.19 (5.64)	<0.01
Blood urea nitrogen (mg/dL)	33.52 (24.73)	31.86 (23.22)	52.09 (32.28)	<0.01
Creatinine (mg/dL)	1.71 (1.43)	1.56 (1.21)	3.37 (2.32)	<0.01
Glucose (mg/dL)	150.18 (64.04)	149.76 (62.16)	154.88 (81.96)	0.04
Calcium (mg/dL)	8.06 (0.97)	8.09 (0.96)	7.73 (1.00)	<0.01
Phosphate (mg/dL)	3.82 (1.48)	3.73 (1.35)	4.88 (2.26)	<0.01
Hematocrit (%)	34.16 (7.18)	34.34 (7.14)	32.33 (7.39)	<0.01
Hemoglobin (g/dL)	11.14 (2.38)	11.19 (2.37)	10.57 (2.43)	<0.01
Red blood cell distribution width (%)	15.69 (2.43)	15.64 (2.40)	16.28 (2.66)	<0.01
Platelet count (mm-3)	214.86 (115.23)	217.68 (114.16)	183.38 (122.30)	<0.01
White blood cell count	14.83 (9.41)	14.72 (9.26)	16.00 (10.85)	<0.01
International Normalized Ratio (INR)	1.54 (0.89)	1.52 (0.87)	1.77 (1.07)	<0.01
Lactate (mg/dL)	2.96 (2.85)	2.85 (2.70)	4.10 (3.98)	<0.01
Albumin (g/dL)	2.68 (0.65)	2.70 (0.65)	2.52 (0.64)	<0.01
Bilirubin total (mg/dL)	1.19 (2.28)	1.10 (2.02)	2.20 (4.06)	<0.01
Alanine aminotransferase (mU/mL)	119.50 (482.86)	103.28 (393.08)	300.06 (1036.68)	<0.01
Aspartate aminotransferase (mU/mL)	197.53 (871.63)	170.68 (737.47)	496.46 (1752.15)	<0.01
Alkaline phosphatase (U/L)	108.24 (98.33)	106.27 (92.79)	130.22 (144.84)	<0.01
Lactate dehydrogenase (U/L)	375.66 (265.25)	373.27 (257.82)	402.28 (336.23)	<0.01
**Vital signs, mean (SD)**				
FiO_2	0.62 (0.14)	0.61 (0.14)	0.63 (0.16)	<0.01
Heart rate (bpm)	93.43 (18.63)	93.17 (18.55)	96.40 (19.29)	<0.01
Respiratory rate (min-1)	21.10 (5.46)	20.94 (5.42)	22.86 (5.66)	<0.01
SpO_2 (%)	96.85 (5.23)	96.86 (5.29)	96.77 (4.54)	0.64
Systolic blood pressure (mmHg)	111.85 (18.16)	112.00 (18.23)	110.13 (17.34)	<0.01
Diastolic blood pressure (mmHg)	60.76 (11.64)	60.98 (11.61)	58.31 (11.61)	<0.01
Mean Arterial Pressure (mmHg)	75.00 (12.74)	75.20 (12.79)	72.74 (11.95)	<0.01
Temperature ©	36.90 (0.90)	36.91 (0.89)	36.79 (1.02)	<0.01
**Medication use, n (%)**				
Any vasopressor	4,123 (46.7)	3,553 (43.9)	570 (78.4)	<0.01
Dopamine	292 (3.3)	252 (3.1)	40 (5.5)	<0.01
Dobutamine	171 (1.9)	143 (1.8)	28 (3.9)	<0.01

Norepinephrine	2,964 (33.6)	2,578 (31.8)	386 (53.1)	<0.01
Phenylephrine	716 (8.1)	600 (7.4)	116 (16.0)	<0.01
Epinephrine	329 (3.7)	249 (3.1)	80 (11.0)	<0.01
Vasopressin	1034 (11.7)	828 (10.2)	206 (28.3)	<0.01
Milrinone	29 (0.3)	25 (0.3)	4 (0.6)	0.45
Heparin	384 (4.4)	344 (4.2)	40 (5.5)	0.14
