# Supplementary figures and images for: High resolution data modifies intensive care unit dialysis outcome predictions as compared with low resolution administrative data set
Source: PLOS Digit Health. 2022 Oct 11;1(10):e0000124. doi: 10.1371/journal.pdig.0000124 (PMC9931257; doi:10.1371/journal.pdig.0000124)

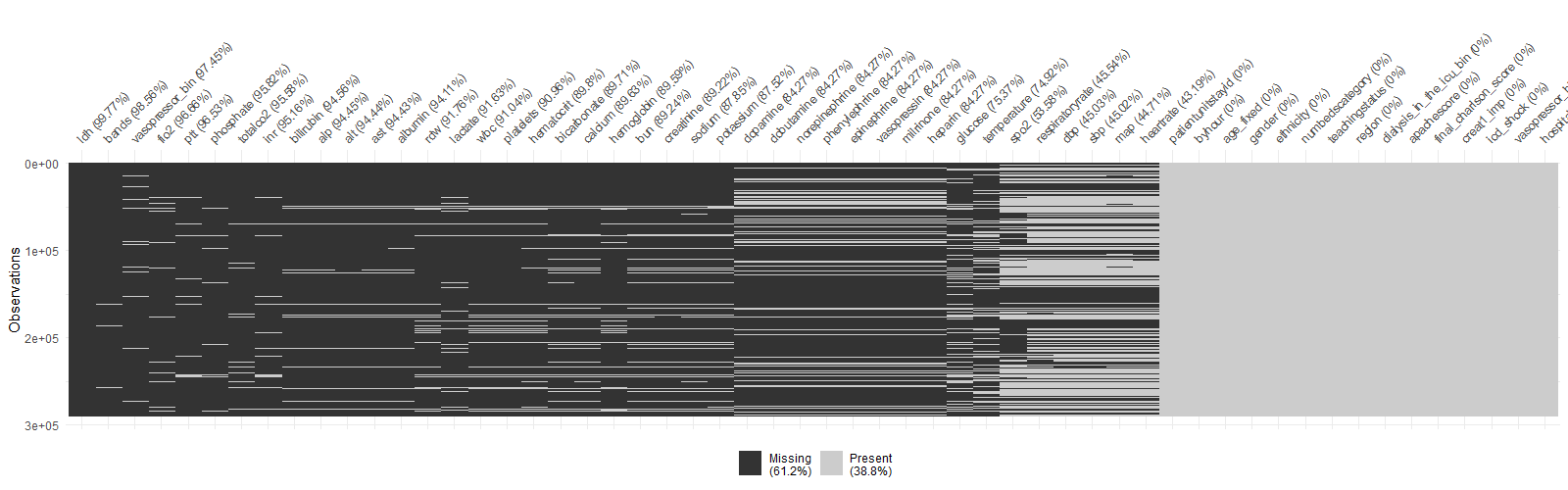

Supplement: S1 Fig — (TIF) [file pdig.0000124.s002.tif]
