# Supplementary material for: High resolution data modifies intensive care unit dialysis outcome predictions as compared with low resolution administrative data set
Source: PLOS Digit Health. 2022 Oct 11;1(10):e0000124. doi: 10.1371/journal.pdig.0000124 (PMC9931257; doi:10.1371/journal.pdig.0000124)
Supplement: Attachment — Submitted filename: Response to reviewers.docx [file pdig.0000124.s003.docx]

Gilles Guillot

Academic Editor

PLoS Digital Health

May 2, 2022

Dear Dr. Guillot,

**SUBJECT:** Submission of revised manuscript PDIG-D-21-00095 “High resolution data modifies intensive care unit dialysis outcome predictions as compared with low resolution administrative data set”

Thank-you for the email dated March 7, 2022 enclosing the reviewer comments for our manuscript. The reviewer comments were very insightful and we appreciate the opportunity to improve the quality of our work. We have carefully reviewed the comments and have amended the manuscript accordingly. We have enclosed our responses to each comment below in red, and have included the specific amendments made to the manuscript with each response.

Our financial disclosure statement is now ammended as follows Research reported in this publication was supported by the National Institute of Health grants

T32DK007527 (ERG) and NIBIB R01EB017205 (LAC). The funders had no role in study design, data collection and analysis, decision to publish, or preparation of the manuscript.

We hope that our revised manuscript and enclosed our accompanying responses will now make our manuscript acceptable for publication in the PLOS Digital Health journal. Each named author has substantially contributed to conducting the underlying research and drafting this manuscript. The authors have no conflicts of interest, financial or otherwise, to declare. Please address all correspondence concerning this manuscript to Dr. Miguel Ángel Armengol de la Hoz at maarmeng@mit.edu. We look forward to hearing from you soon.

Sincerely,

Miguel Ángel Armengol de la Hoz, PhD / Research Affiliate

MIT - Institute for Medical Engineering and Science

Laboratory for Computational Physiology

MIT, E25-235- 53 Carleton St.

Cambridge, MA 02139

**Journal Requirements:**

1. Please amend your detailed Financial Disclosure statement. This is published with the article, therefore should be completed in full sentences and contain the exact wording you wish to be published.

i). State the initials, alongside each funding source, of each author to receive each grant.

ii). State what role the funders took in the study. If the funders had no role in your study, please state: “The funders had no role in study design, data collection and analysis, decision to publish, or preparation of the manuscript.”

We apologize for these errors. We have amended the manuscript in line with these requirements. The Financial Disclosure section of the manuscript has been updated with these recommendations (page 19):

***Financial Disclosure***

*Research reported in this publication was supported by the National Institute of Health grants*

*T32DK007527 (ERG) and NIBIB R01EB017205 (LAC). The funders had no role in study design, data collection and analysis, decision to publish, or preparation of the manuscript.*

2. Please update your Competing Interests statement. If you have no competing interests to declare, please state: “The authors have declared that no competing interests exist.”

We apologize for not including this in our initial manuscript. This has been added to the manuscript (page 20):

***Competing Interests***

*The authors have declared that no competing interests exist.*

3. We have noticed that you have uploaded supporting information but you have not included a list of legends. Please add a full list of legends for all supporting information files (including figures, table and data files) after the references list.

We apologize for this error. The list of legends including all figures, tables and data files has been updated with this required information (page 26).

**Reviewer #1:**

1. For the high-resolution covariates, what was the time range used to predict mortality? e.g. was the median of first hour values after ICU admission used? or was the median of first day values used?

Thanks a lot for your inquiry. We calculated the median of the values from the first 24 hours of admission. A safety window of 6 hours prior to admission was included so we could capture data that was charted with an incorrect timestamp, an issue we have previously identified in this dataset.

2. Please specify if normalization of variables was used before doing logistic regression.

We apologize for this omission in our methodology. Normalization of the variables was not performed prior to the logistic regression analyses. The manuscript has been updated to include this clarification in the methods section and now includes the following statements (page 19):

*Normalization of the variables was not performed prior to performing the logistic regression analysis.*

*Both the low resolution (listed in the Base Covariates section) and high-resolution (listed in the High-Resolution Covariates section) covariates described above were used, and were not normalized prior to analysis.*

3. The p-values in Table 1 are comparison between no-dialysis and with-dialysis in each cohort. Please indicate this in the caption. Also, if the caption could detail the test methods used, that could be better.

Thank-you for the comment, we apologize that this was not made clear. The caption for table 1 and the methods section have been updated to reflect this suggestion. The table 1 caption is now as follows:

*Table 1. The baseline characteristics of the patients in the eICU and NIS cohorts. The patients receiving dialysis and those not receiving dialysis from each cohort are compared using the Student’s independent t-test of Wilcoxon Rank Sum test for continuous variables, and the Chi-Square test for categorical variables.*

The methods section is updated as follows (page 17):

***Cohort Selection***

*Comparable cohorts of patients with sepsis who required mechanical ventilation were created in the NIS and eICU databases. The details of cohort selection for each database can be found in Figures 1 and 2. The baseline characteristics of the patients in both the NIS and eICU cohorts is shown in Table 1. The patients from each cohort are compared by use of dialysis using either the Student’s Independent t-test or the Wilcoxon Rank Sum depending on normality for continuous variables, and the chi-square analysis for categorical variables.*

4. Is it necessary to evaluate how good the model fitting was? The experiment shows that no evidence supports that 'dialysis' in the high-res model is significant, while it was in the low-res models. But why would we trust the high-res model? Was it over-fitted? It had much more variables.

Thanks a lot for your question. The main objective of our hypothesis would not require training and validating a model but only fitting it in order to address the effect of the main variables for predicting the outcomes. The additional variables included in the high resolution model were selected based on clinical significance, determined *a priori*. The high resolution model had a superior area under the curve (AUC) in comparison to the low resolution model. The Area under the curve (AUC) of the High Resolution Model is 0.82, and 0.73 for the Low Resolution Model. Based on these points, we feel that the high resolution model is reliable and superior to the low resolution model.

5. It seems that the experiment did not intent to make the populations of the two cohorts similar. Table 1 did not compare between the two cohorts. e.g. the male percentage, the mortality rate and shock in two cohorts seem to be different. There was no difference in the percentage of dialysis (with proportion test). NIS cohort and eICU cohort had similar percentage of cases with dialysis; NIS cohort had higher mortality rate, and shock. Would the population difference affect the model comparison?

Thank you for your comment and question. We did indeed attempt to make the cohorts similar, by using the same inclusion and exclusion criteria. Thus while there may have been slight differences in the composition of the cohorts and outcomes, we were attempting to make similar cohorts and succeeded in doing so. It would be very difficult or impossible to find two other large nationwide US datasets that would have the data needed for such a comparison, and we make no assumptions of generalizability of our findings to other cohorts or countries.

Generally, I agree with the authors' claim that more detailed clinical variables would better help to answer clinical questions. With more useful variables being added to the model, it is likely that previously significant variables would be less important. As the authors said, this is "a thought experiment and proof of concept" study. More experiments and comparison in the future would better support the claim.

Thanks for your comment. We agree that additional studies examining our hypothesis would strengthen the overall findings and understanding of high-resolution and low-resolution databases. We have amended our final conclusions to reflect this comment as follows (page 16):

*Further studies examining the outcome of clinical research questions using both high and low resolution datasets should be performed in the future to further evaluate the impact of dataset granularity on the results of observational studies.*

**Reviewer #2:**

Predictive models in critical care have been around for decades. Almost all of the major ones use information extracted electronically or manually from Electronic Medical Record systems (EMR). It is rare to see an observational study in critical care that uses administrative data, with the only major caveat being that because of the COVID pandemic there were studies that relied on data not gleaned from an EMR.

Thank-you for your comments. While we agree that in recent years, observational studies in critical care often use data extracted from electronic medical record systems, studies using administrative databases are still very common in the literature. Two recently published editorials, “Administrative Databases Are Here to Stay” by Barba (2021) and “Administrative Medical Databases for Clinical Research: The Good, The Bad, and The Ugly” by Rabinstein (2018), both highlight the importance of studies that use administrative databases in critical care, but acknowledge the inherent limitations of these types of analyses. Furthermore, we disagree that it is rare to find studies in critical care on topics other than the COVID-19 pandemic that rely on administrative databases. We have identified a number of studies published within the past year using the NIS database alone, including the following publications (1–5):

1.	Elkaryoni A, Darki A, Bunte M, Mamas MA, Weinberg I, Elgendy IY. Palliative Care Penetration Among Hospitalizations with Acute Pulmonary Embolism: A Nationwide Analysis. J Palliat Care [Internet]. 2022 Feb 9 [cited 2022 Apr 13];8258597221078389. Available from: http://www.ncbi.nlm.nih.gov/pubmed/35138196

2.	Kaewput W, Thongprayoon C, Dumancas CY, Kanduri SR, Kovvuru K, Kaewput C, et al. In-hospital mortality of hepatorenal syndrome in the United States: Nationwide inpatient sample. World J Gastroenterol [Internet]. 2021 Dec 7 [cited 2022 Apr 13];27(45):7831–43. Available from: http://www.ncbi.nlm.nih.gov/pubmed/34963745

3.	Nemeh C, Dongarwar D, Oduguwa E, Salihu HM, Barrantes Perez J. Clinical Outcomes of Noninvasive Ventilation in Asthma, Bronchiectasis, and Other COPD. South Med J [Internet]. 2021 [cited 2022 Apr 13];114(12):766–71. Available from: http://www.ncbi.nlm.nih.gov/pubmed/34853852

4.	Shah H, ElSaygh J, Raheem A, Yousuf MA, Nguyen LH, Nathani PS, et al. Utilization Trends and Predictors of Non-invasive and Invasive Ventilation During Hospitalization Due to Community-Acquired Pneumonia. Cureus [Internet]. 2021 Sep [cited 2022 Apr 13];13(9):e17954. Available from: http://www.ncbi.nlm.nih.gov/pubmed/34660142

5.	Sharma A, Nguyen P, Taha M, Soubani AO. Sepsis Hospitalizations With Versus Without Cancer: Epidemiology, Outcomes, and Trends in Nationwide Analysis From 2008 to 2017. Am J Clin Oncol [Internet]. 2021 [cited 2022 Apr 13];44(10):505–11. Available from: http://www.ncbi.nlm.nih.gov/pubmed/34342290

It has been well documented that using an administrative database is less precise than using EMR data. Perhaps the novelty in the manuscript by Ziegler et al lies in showing that it’s not just general mortality predictions that are less precise when developed from administrative data, but that clinical studies could have considerably different results based on the data source (administrative vs. EMR). But to effectively make that case necessitates looking at more than one effect-outcome analysis. Thus, my major complaint about this manuscript is that it needs to incorporate more analyses to make its case. Certainly with the NIS and eICU datasets this is not too difficult to carry out.

Thank-you for the insightful comments. We agree that clinical studies can have widely variable results depending on the data source, as statistical modeling is dependent on the quality of data, the data source, as well as the covariables included. Our current study highlights this point as we have shown that the prediction in our models had significantly different results once the clinical variables from the high resolution model were used compared to the low resolution variables. We also agree that more analyses are required to fully support our findings that high resolution data allows for improved ability to control for confounders in prediction models compared to when using low resolution data. However, we do not feel that additional analyses examining the current research question would be feasible to incorporate into one succinct manuscript. Our group is in the process of conducting further research to reinforce this study’s current findings. We have begun a study examining the effect of the use of arterial catheterizations among patients on mechanical ventilation using both the low resolution data set (NIS) and high resolution data set (eICU). A prior study by Dr. Celi and colleagues (Hsu et al. 2015) using a high resolution dataset showed that arterial catheterization was not associated with a difference in 28-day mortality among hemodynamically stable patients requiring mechanical ventilation. This future project will serve to validate the prior findings in another high resolution dataset, and to compare the outcomes with a low resolution dataset.

The authors seem to disregard databases such as APACHE and Project IMPACT, as well as national repositories (ICNARC, ANZICS) that have provided hundreds of clinical studies. These data sources need to be discussed in the manuscript, as they are similar to the eICU data repository in many respects.

Thanks for your comments. We have updated our discussion section to reflect the importance of these databases and their similarities to the eICU database as follows (page 13):

*In contrast to administrative datasets and disease registries, more recently developed high resolution databases such as eICU, were created with its use for clinical research as a primary objective. Globally, there are several other examples of commercial and non-commercial high resolution databases and national repositories in the field of critical care that are currently used for medical research. In the United States, examples include the Veterans Affairs patient database, the MIMIC-II database and the APACHE and Project IMPACT databases (25,40–42). In the United Kingdom, the Intensive Care National Audit and Research Center (ICNARC) provides high resolution data from national repositories, and similarly, the Australian and New Zealand Intensive Care Society also curates a large database from patient ICU stays (43). These high resolution datasets are well designed and structured to answer clinical questions. They also contain vast amounts of clinical data such as patient vital signs and laboratory values captured from various electronic sources in hospitals(24). These datasets are increasingly being used due to the powerful data available to address clinical research questions, and have provided data to support hundreds of studies in recent years.*

There is virtually no information given on the multivariable logistic regression model. In fact, there’s no Methods section at all. Advising readers to go to github rather than present the gist of their methodology in the paper is regretful.

Thanks a lot for your feedback, the methodology section describing our high resolution model was expanded now including (page 19)*:*

*A multivariate logistic regression model (estimated using maximum likelihood) was fitted to predict in-hospital mortality for patients admitted to the ICU with sepsis while requiring mechanical ventilation was performed with the eICU cohort. Standardized parameters were obtained by fitting the model on a standardized version of the dataset. A typical predictor is fitted for binomial families, the response is specified as a factor where the first level denotes failure and all others success. Both the low resolution (listed in the Base Covariates section) and high-resolution (listed in the High Resolution Covariates section) covariates described above were used, and were not normalized prior to analysis. The variables included in the high resolution model were determined a priori. In order to address collinearity of the model, a correlation plot was generated to study all the interaction between the model variables (Figure 3).*

**Reviewer #3:**

Generally, the article is well written, cogent, and reasonable. However, I found the order to be confusing. The article jumped from introduction to results and then to discussion. It presented Materials and Methods at the end. I was left to read a discussion about a set of models I hadn't yet been introduced to. I think the paper would be greatly improved if it followed a more traditional Introduction, Methods, Results, and Discussion structure. It is also worth noting that there were a large number of comma errors in the paper which distracted me from the content of the paper. These should be corrected.

Thank you for the comment. The Methods section can be moved up, however this is the suggested organization of the journal. We are happy to move the Methods section up if it pleases the reviewer and the editor of the journal. We were merely following the suggested order by the journal. Please see the Submission Guidelines at https://journals.plos.org/digitalhealth/s/submission-guidelines

In the introduction, I struggled with a few points. For example, I didn't understand what the authors meant by "unselected patient samples" (line 97). Additionally, the authors refer to "outcomes" (link 99) but I think this deserves greater clarification. What is the difference between "accuracy and validity" (line 104)? It also isn't clear where "medical registries" fit into this dynamic between low resolution clinical databases vs EHRs. Moreover, it isn't entirely clear to me what the authors mean when they distinguish between low resolution and high resolution in the paper. This must be made more explicit, with consistent usage throughout the paper.

Thanks for the comments. We clarified the wording of “unselected patient samples” and “outcomes” as follows (page 4-5):

*Administrative databases, such as the Nationwide Inpatient Sample (NIS), provide large patient samples and include valuable and reliable information such as patient demographics, diagnostic coding of primary and secondary diagnoses, procedures performed, length of hospitalization and discharge status (ie. Discharge, death, transfer to another facility) (7).*

We have removed the term “validity” from line 104.

We have clarified that medical registries are often considered low resolution datasets with the following (page 5):

*Medical registries are another type of low resolution data, which include health services registries, product registries and disease registries, and are also important sources of data for medical research and epidemiological studies (11–13).*

We have clarified the distinction between high resolution and low resolution databases in the introduction section with the following amendments (pages 4 and 6):

*Low resolution databases are data sets that lack granular and detailed clinical data, and often contain pre-specified types of information, such as patient demographics, diagnoses, hospital information as well hospital admission and discharge information*

*More recently, the widespread use of EHRs has provided access to large amounts of clinical data (21,22). The EHR data with the integration of clinical monitoring systems have allowed for the development of modern databases containing high fidelity clinical information. These datasets with highly granular and detailed clinical data can be considered as high resolution databases. High resolution databases often draw information from EHRs including patient monitoring parameters and physiological data, patient care flow sheets, laboratory values, procedures performed as well as the temporal trends throughout a patient’s clinical course. Databases such as the eICU Collaborative Research Database (eICU) and the Medical Information Mart for Intensive Care (MIMIC) are examples of high resolution databases(23,24)*

Finally, I found the use of the term "causal inference techniques" (line 136) to be insufficiently vague.

Thanks for the comment. We have removed this term and clarified as follows (page 6):

*Statistical models used to analyze data in non-randomized trials are limited by numerous factors.*

In the Results, it wasn't clear to me how the p-values were calculated. I didn't see sufficient documentation in the methods section to find an answer. This will need to be clarified. Additionally, the code repository should be linked directly (line 181). I was unable to find the exact code in the link provided.

Thanks for the comment, we apologize that this was not included in our initial manuscript. We have provided further explanation of how the p-values were calculated in the methodology section (page 17):

*All statistical analyses in this study were performed assuming a two-sided alpha level of 0.05.*

Github url was updated and the latest commits of the projects were pushed to the repository. The code repository was also made public. In the results sections you can now find the following text (page 11):

*The authors provide open access to all their data extraction, filtering, data wrangling, modeling, figures and tables, code, and queries on https://github.com/theonesp/hr_vs_lr_repos.*

In the Discussion section, I think the core argument of the paper is hidden in line 231. It is my opinion that the paper would benefit from making this more explicit earlier in the paper -- perhaps in the introduction. As further evidence that this is secretly the core argument of the paper, I point to line 254, where the authors clearly document the value of the paper as a contrast between data from EHRs vs administrative databases. But that perspective isn't forcefully presented to the reader, which weakens its value.

Thanks for the constructive feedback. We have updated our introduction to emphasize the argument that low resolution models lack the ability to adjust for clinical confounders that can be accounted for in high resolution models. The text of the introduction is clarified as follows (page 6-7):

*Statistical models used to analyze data in non-randomized trials are limited by numerous factors. One of the major barriers to accuracy of modeling is the presence of residual confounding not accounted for by the study design (29). Specifically, in the intensive care unit, administrative datasets often lack detailed clinical information about severity of illness, laboratory investigations, and vital signs, as well as time-stamped interventions, which are likely important confounders not accounted for in the models (30). Conversely, the use of robust databases that incorporate clinical and bedside monitoring data might improve modelling accuracy due to the ability to control for additional clinical variables and confounders, that are not available with low resolution datasets. The aim of this experiment is to compare the ability to adjust for confounding between a low resolution large national administrative database and a high resolution large multi-center EHR database examining the same clinical question.*

We have also updated the initial paragraph in the discussion to strengthen our main argument (page 12):

*These results demonstrate that after the addition of the high resolution clinical variables to the model, the results of the analysis changed significantly. This highlights the importance of the granular clinical data within the high resolution model, as the low resolution model did not account for these important clinical confounders.*

In the Methods section, I don't know if "specificity" is the best word choice in line 267. In line 273, I don't think the authors are being entirely clear when they write "and is designed to capture approximately >95% of all inpatient care." How can the database simultaneously "capture" 20% and 95% of inpatient cases?

Thank you for your comment and apologize that this was not clear in our initial submission. The NIS captures 20% of hospital admissions at each US institution, however it is designed in a sample survey technique so that analyses with this dataset will approximate 95% of inpatient care. The missing 5% comes from care delivered at prison hospitals and a few other non traditional hospital types. We have changed the wording of the description of the NIS database to (page 16):

*It captures approximately 20% of all inpatient hospitalizations and is designed to approximate >95% of all inpatient care (prison hospitals and non-traditional hospitals are excluded).*

As for the word specificity, we have changed it to certainty.

On line 316, the authors write that missing data were imputed using "forward-backward filling imputation method." This will need to clarified. More specifically, under which circumstances did they use forward filling and which circumstances did they use backward filling? For what variables? What percentage of observations were affected? Why do they think this was appropriate over other methods?

Thanks a lot for your question. For continuous variables we are using backward and forward fill for each variable individually grouping by patientunitstayid, which is our unit of analysis, the patient identifier. Forward filling means to fill missing values with previous data available for a given patient. Backward filling means to fill missing values with the next data point available for a given patient. So what our filling function does when a cell is missing a value is first it tries to find the next available cell in the downer rows and when there are no downer rows with information it fills with the next available cell in the upper rows. If there is no data available in the downer and upper rows for that given patient, the cell will remain empty. For binary columns we did not perform back-forward fill. If there is a cell with missing data, it means they did not receive a dose, so we replace it with 0. We also included a missingness plot in the supplementary material addressing the missing percentage per variable.

We have amended our manuscript reflecting this as follows (page 18-19):

*Missing data for the high-resolution model continuous variables were imputed using a forward-backward filling imputation method. Forward filling means to fill missing values with previous data available for a given patient. Backward filling means to fill missing values with the next data point available for a given patient. The function first attempts to fills the data point with the backward method if a datapoint is available, and if not available, then with the forward method. For binary data, missing data was treated as a 0. Missing data for the high-resolution binary medication variables were treated as not administered. The plot of missingness for each variable is shown in supplemental material S2.*

Finally, I think that the authors need to convince the reader that they simply haven't introduced colliders or redundant (i.e. collinear) variables into the models to achieve the demonstration of confounding. If they simply added collinear variables, then, of course, we would see a drop in the odds ratio. How do we know that this isn't the effect in question?

Dear reviewer, we appreciate your suggestion and appended a figure for collinearity to address this issue. It is now included as figure 3 in our manuscript. We also added the comment (page 19):

*In order to address collinearity a correlation plot was generated to study all the interaction between the model’s variables.*
