# Supplementary material for: High resolution data modifies intensive care unit dialysis outcome predictions as compared with low resolution administrative data set
Source: PLOS Digit Health. 2022 Oct 11;1(10):e0000124. doi: 10.1371/journal.pdig.0000124 (PMC9931257; doi:10.1371/journal.pdig.0000124)
Supplement: Attachment — Submitted filename: Response to reviewer comments.docx [file pdig.0000124.s004.docx]

**Response to reviewer comments: PDIG-D-21-00095 “High resolution data modifies intensive care unit dialysis outcome predictions as compared with low resolution administrative data set” (Ziegler, J et al. )**

Journal Requirements: 1. Please review your reference list to ensure that it is complete and correct. If you have cited papers that have been retracted, please include the rationale for doing so in the manuscript text, or remove these references and replace them with relevant current references. Any changes to the reference list should be mentioned in the rebuttal letter that accompanies your revised manuscript. If you need to cite a retracted article, indicate the article's retracted status in the References list and also include a citation and full reference for the retraction notice.

We have carefully reviewed our reference list to ensure that it is complete and correct. We have also reviewed the reference list in detail, and have not identified any retracted papers to the best of our knowledge. We have not made any changes to the reference list. If the editor identifies any retracted articles in our reference list that we have missed, we would happily remove the applicable citations from our manuscript.

Reviewer #1: One more suggestion. Please add one or two sentences to describe the points of Figure 3 (i.e. what do the authors want to tell the readers?). Does it mean only a few variables are high correlated? or all pairwise correlations are under a certain threshold? The plot is just a tool to check, but not a (direct) tool that reduce collinearity.

Thanks for the comment. We have added a description of the correlation plot (Fig 3) in the results section. The additional text is as follows:

The correlation plot (Fig 3) shows that overall, there was not significant correlation between the majority of the high resolution variables. The pairs of variables that did show high correlation were hemoglobin and hematocrit, AST and ALT, mean arterial pressure and diastolic blood pressure, and mean arterial pressure and systolic blood pressure.
